# Four-week neoadjuvant 5% imiquimod prior to Mohs micrographic surgery for melanoma in situ: A prospective pilot study

**DOI:** 10.1016/j.jdin.2026.03.010

**Published:** 2026-03-26

**Authors:** David T. Harvey, David I. Latoni, Yiwen Helen Li, Dipti Anand

**Affiliations:** a Anne Arundel Dermatology, Newnan, Georgia; b Department of Dermatology, Emory University School of Medicine, Atlanta, Georgia; c Department of Dermatology, University of Puerto Rico School of Medicine, San Juan, Puerto Rico; d SkinPath Solutions, Smyrna, Georgia

**Keywords:** 4 weeks, imiquimod, melanoma in situ, MIS, Mohs, neoadjuvant, skin surgery, tumor burden, tumor shrinkage

*To the Editor:* Melanoma in situ (MIS), including lentigo maligna (LM), often develops on chronically sun-damaged head and neck skin. These tumors can extend subclinically beyond visible borders, which makes complete clearance with standard excision difficult. Mohs micrographic surgery (MMS) provides excellent margin control and low recurrence for head and neck melanoma.^1^ However, guideline-recommended margins for LM/MIS (5-10 mm) can be challenging on cosmetically and functionally sensitive sites.^2^ Topical 5% imiquimod has reported clearance rates of ∼70% to 80% for LM/MIS.^3^ It has also been used as an adjunct to surgery.^4^ Neoadjuvant use before MMS is less studied. Prior reports often require prolonged (∼12-week) regimens.^5^ We evaluated whether a shorter, 4-week neoadjuvant course could rapidly debulk MIS/LM before MMS while maintaining oncologic safety.

We enrolled 11 patients with MIS/LM treated at a single private dermatologic surgery practice in Georgia (2024-2025). Patients applied imiquimod 5% once daily, 7 days/week, for 4 consecutive weeks before MMS. Lesions were measured under Wood’s lamp before and after treatment. Length and width were multiplied to estimate surface area. Residual tumor was assessed during MMS with melanoma antigen recognized by T cells 1 immunostaining of frozen sections. For the single case with complete clinical resolution after imiquimod, with no clinically identifiable residual lesion, two to three scout biopsies (3 mm) were obtained from the original site and adjacent clinically normal-appearing skin to assess for residual melanoma. An intention-to-treat analysis included all 11 patients. One patient was lost to follow-up before MMS, but post-treatment clinical findings were recorded. Detailed methods and subject characteristics are in Supplementary Figs 1 and 2, available via Mendeley at https://data.mendeley.com/datasets/3mhvkt52fy/1.

Results showed rapid, substantial debulking with a high histologic clearance rate despite the abbreviated regimen. All 11 patients improved clinically. Mean lesion area decreased 68% (95% confidence interval: 55% to 81%; standard deviation: 19%; *P* < .0001). Ten patients underwent histologic evaluation. Seven (70%) achieved complete histologic clearance. Two (20%) had partial response with residual microscopic melanoma. One (10%) had invasive melanoma identified after treatment. Adverse effects were localized and expected, including erythema, edema, and crusting. No systemic adverse events occurred. No patient discontinued therapy. Among complete responders, 6 cleared in a single MMS stage. One patient had complete clinical resolution after imiquimod with no targetable site for MMS; scout biopsies were negative, and the patient elected close clinical follow-up. Partial responders still showed meaningful debulking. One cleared in 1 stage. One required 3 stages due to residual MIS and a coincident intradermal nevus. In the nonresponder, scout biopsies revealed dermal invasion (Breslow 0.6 mm). The patient was referred for staged excision/slow Mohs. No distant metastasis was noted on surveillance.

This prospective pilot supports a neoadjuvant strategy that pairs brief topical immunotherapy with definitive margin control. The novelty is the 4-week regimen and the magnitude of early debulking (68%), alongside 70% complete histologic clearance and frequent single-stage MMS. Fig 1, *A* and *B* illustrates representative clinical debulking in a patient, showing the lesion before imiquimod and after the 4-week course (immediately pre-MMS). Clinical regression may not exclude residual or invasive disease. Post-treatment biopsy confirmation and definitive surgery remain essential. Limitations include small sample size, single-center design, 1 loss to follow-up, limited follow-up (<5 years), and potential sampling error/skip areas on photodamaged skin.

**Fig 1:**
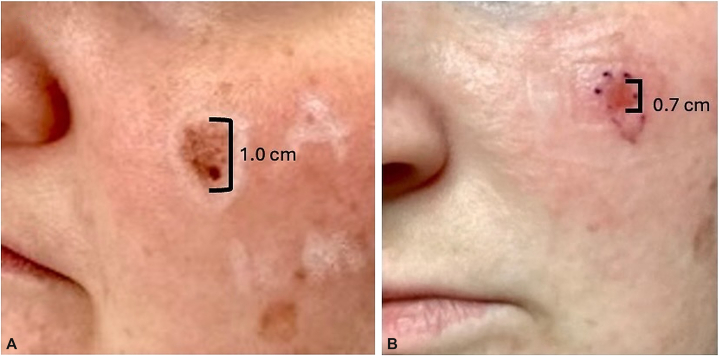
Clinical photos of representative patient. **A,** Original presenting lesion (MIS); (**B**) lesion 4 weeks after treatment with topical imiquimod. *MIS*, Melanoma in situ.

### Declaration of generative AI and AI-assisted technologies in the writing process

During the preparation of this work the authors used ChatGPT v5.2 for proofreading and grammatical improvements. After using this tool, the authors reviewed and edited the content as needed and take full responsibility for the content of the publication.

## Conflicts of interest

None disclosed.
